# Editorial: Agricultural innovation in the age of climate change: a 4.0 approach

**DOI:** 10.3389/fpls.2025.1715677

**Published:** 2025-10-13

**Authors:** Angelo Cardellicchio, Vito Renò, Carmela Rosaria Guadagno, Francesco Cellini, Chiara Amitrano

**Affiliations:** ^1^ Istituto di Sistemi e Tecnologie Industriali Intelligenti per il Manifatturiero Avanzato, Consiglio Nazionale delle Ricerche (CNR-STIIMA), Bari, Italy; ^2^ Botany Department, Science Institute, University of Wyoming, University of Wyoming, Laramie, WY, United States; ^3^ Agenzia Lucana di Sviluppo e Innovazione in Agricultura, Metaponto, Italy; ^4^ Dipartimento di Agraria, Università degli Studi di Napoli Federico II, Napoli, Italy

**Keywords:** precision agriculture, sustainability, deep learning, artificial intelligence, robotics & automation in agriculture

The global agricultural landscape is constantly evolving, with a focus on identifying solutions to feed an increasingly large population, projected to reach 10 billion people by 2050 (UN, Department of Economic, 2024). This challenge arises at a time of increasing risks posed by the current rate of climate change (Kikstra et al., 2022), leading to an increased frequency of extreme weather events, shifting precipitation patterns, and new pest pressures due to ecosystem changes and adaptations.

In this scenario, *Agriculture 4.0* has emerged as a critical adaptation path toward developing resilient, efficient, and sustainable farming practices, characterized by the harmonization of modern technologies such as artificial intelligence (Cardellicchio et al., 2025), collaborative robotics (Reina et al., 2016), big data, and distributed smart sensors (Akhter and Sofi, 2022). This Research Topic, *Agricultural Innovation in the Age of Climate Change: A 4.0 Approach*, combines five different research areas to showcase the breadth and depth of such innovation.

## Robotics and perception in agriculture 4.0

The contributions demonstrate that Agriculture 4.0 is a feasible and effective approach that encompasses various applications and scenarios. Specifically, Wang et al. investigated one of the most relevant and foundational elements of Agriculture 4.0, that is, the advancements in robotics required to enable precision agriculture. This requires overcoming several issues related, for example, to terrain estimation (Vulpi et al., 2021). Specifically, the authors introduced a fuzzy backstepping controller, harmonizing the trailer’s trajectory with the tractor’s, and ensuring higher precision in operations such as seeding and fertilization, thus improving operational costs and overall yield. A complementary contribution involving AI was proposed by Divyanth et al., who proposed a system based on single-stage object detectors to address post-harvest challenges in pathogen detection in potatoes, identifying potato eyes in a high-throughput scenario, and automating a traditionally labor-intensive process for disease management.

## Accessibility and user engagement

Another relevant aspect is accessibility to modern technology, which is often a constraint when working with the main users of the proposed advancements: breeders and farmers. To this end, Won et al. demonstrated the potential of using standard, low-cost devices, such as smartphones, for nondestructive crop monitoring. Specifically, the authors established a meaningful correlation between the lettuce leaf color, captured in the RGB color space, and the plant’s fresh weight across different nutritional treatments, thus opening the door to low-cost tools for crop health assessment and yield prediction. In another approach to output optimization, Cao et al. proposed a simulated environment to enhance greenhouse strawberry yields by optimizing the patterns of honeybee pollination. Their work revealed that specific interplanting strategies, along with staggered planting times, can significantly improve cross-pollination and fruit weight by mitigating competition among flowers for pollinators. This demonstrates how numerical models can be used to understand and manage complex biological systems and provide valuable and effective guidance for breeders.

## Economics and societal challenges

Finally, Zhang et al. addressed a related issue that can be solved by adopting modern digital solutions. The authors used data analytics and intelligence to address the economic volatility that is inherently built into the agricultural sector, and that is of special interest in dynamic scenarios such as the ones we are currently living in. This research focused on predicting agricultural commodity prices, using a BiLSTM-based hybrid model to provide highly accurate price forecasts for key commodities, including ginger, garlic, pork, and soybean futures. This predictive power can be an incredibly useful asset for stabilizing the market, thus optimizing the supply chain and supporting political decision-makers in their policymaking.

Together, all these different studies paint a compelling picture of what Agriculture 4.0 can offer to the community, highlighting how digital innovation offers a diverse toolkit for addressing long-standing and emerging challenges. The research community operates with a wide range of applications, from soil management to the precise control of field robots, focusing on intelligent automation, yield optimization via accessible devices, simulation-based optimization and adaptation, and the data-driven forecasting of exogenous and economic trends. Therefore, these contributions underscore the transformative potential of Agriculture 4.0 in building a more effective, resilient, and sustainable agricultural landscape, tailored to navigate the challenges and complexities we will face in this century.
